# Astragaloside IV alleviates lung inflammation in *Klebsiella pneumonia* rats by suppressing TGF-β1/Smad pathway

**DOI:** 10.1590/1414-431X2023e12203

**Published:** 2023-07-21

**Authors:** Lei Li, Jie Guan, Rongjun Lin, Fang Wang, Hui Ma, Chenggang Mao, Xingqing Guo, Zhenghai Qu, Renzheng Guan

**Affiliations:** 1 Department of Pediatrics, The Affiliated Hospital of Qingdao University, Qingdao, China; 2 Department of Neurology, Qingdao Hiser Hospital Affiliated to Qingdao University (Qingdao Traditional Chinese Medicine Hospital), Qingdao, China

**Keywords:** Lung inflammation, TGF-β1/Smad pathway, Astragaloside IV, K. pneumoniae, Pharmacology

## Abstract

Astragaloside IV is a biologically active substance derived from the traditional Chinese medicine *Astragalus mambranaceus* Bunge, and has antioxidant, anti-inflammatory, and anti-apoptotic properties. In this study, we aimed to investigate the effects of astragaloside IV on *Klebsiella pneumonia* rats and the underlying mechanisms. *Klebsiella pneumoniae* (*K. pneumoniae*) rats were treated with different dosages of astragaloside IV (5, 10, and 20 mg/kg) by intragastric administration. The levels of pro-inflammatory cytokines interleukin (IL)-6, IL-1β, and tumor necrosis factor (TNF)-α in bronchoalveolar lavage fluid (BALF) were determined. Pathological changes of lung tissue were inspected by HE staining. The expression of transforming growth factor (TGF)-β1 in lung tissue was determined with immunohistochemistry, and the expression levels of TGF-β1, p-Smad2/Smad2, p-Smad3/Smad3, IκBα/p-IκBα, and p65/p-p65 in lung tissue were determined by western blot. The mechanism was further investigated with TGF-β1 inhibitor SB-431542. Astragaloside IV reduced the elevated levels of pro-inflammatory cytokines caused by *K. pneumoniae* and improved lung tissue damage in a dose-dependent manner. Astragaloside IV also decreased the expression of TGF-β1/Smad signaling pathway-related proteins and decreased the protein levels of inflammation-related p-IκBα and p65 in lung tissues induced by *K. pneumoniae*. Additionally, it was found that the effects of 20 mg/kg astragaloside IV were similar to SB-431542, which could improve pulmonary fibrosis induced by *K. pneumoniae*, decrease the levels of TGF-β1/Smad signaling pathway-related proteins in lung, and reduce inflammation at the same time. Astragaloside IV could alleviate the inflammation of rat pneumonia induced by *K. pneumoniae* through suppressing the TGF-β1/Smad pathway.

## Introduction

*Klebsiella pneumoniae* (*K. pneumoniae*) is a pathogenic Gram-negative capsulated bacterium that causes urinary tract infections, pyogenic liver abscess, and pneumonia, and has become one of the leading causes of hospital-acquired infections (1,2). Researchers have increasingly focused on pneumonia caused by this clinically relevant bacterium in murine models in order to find new ways to deal with the disease (3,4).

Transforming growth factor β1 (TGF-β1) is a pleiotropic cytokine that is known to have functions in immunomodulation, angiogenesis, and extracellular matrix formation (5,6). TGF-β1 exhibits its biological functions through Smad-dependent and -independent pathways (7). By the well-known TGF-β/Smad pathway, TGF-β1 can activate Smad2 and Smad3 to translocate into the nucleus and initiate gene transcription, while being negatively regulated by Smad7 (8,9). A number of studies have already shed light into the role of TGF-β/Smad signaling pathway in lung injury (10,11).

Astragaloside IV is a biologically active substance derived from the traditional Chinese medicine *Astragalus mambranaceus* Bunge and has antioxidant, anti-inflammatory, and anti-apoptotic properties (12-14). In recent years, astragaloside IV has received attention in the prevention of respiratory diseases, such as pulmonary fibrosis (15) and asthma (16). In these studies, researchers found that astragaloside IV treatment is not toxic to lung tissue, and inhibits oxidative stress and inflammatory response to reduce the progression of pulmonary fibrosis. There has been much research that related astragaloside IV with TGF-β/Smad signaling pathway inhibition in various disease models, including pulmonary disease (17-19). In recent years, astragaloside IV has been successfully applied in the treatment of pneumonia induced by virus infections and smoke, though the mechanisms discovered were quite different (20,21). However, there is lack of data regarding the effects of astragaloside IV on *K. pneumoniae*.

This study aimed to assess the role of astragaloside IV in *K. pneumoniae* and the signaling pathway, which hopefully might help the development of novel therapy for this important hospital-acquired infection.

## Material and Methods

### Animals

Ninety Sprague Dawley male rats weighing 200-220 g were purchased from Jinan Pengyue Experimental Animal Breeding Corporation (SCXK(Lu)20190003, China). Rats were maintained in standard condition at 23±2°C and 55±5% humidity on a 12-h light/dark cycle. Rats were provided with food and water *ad libitum*. All the animal experiments followed the guidelines of the National Institutes of Health (NIH, Pub. No. 85-23, revised 1996) and were approved by the Animal Use and Care Committee of The Affiliated Hospital of Qingdao University (QYFYWZLL26490).

### *K. pneumoniae* rats

**Figure 1:**
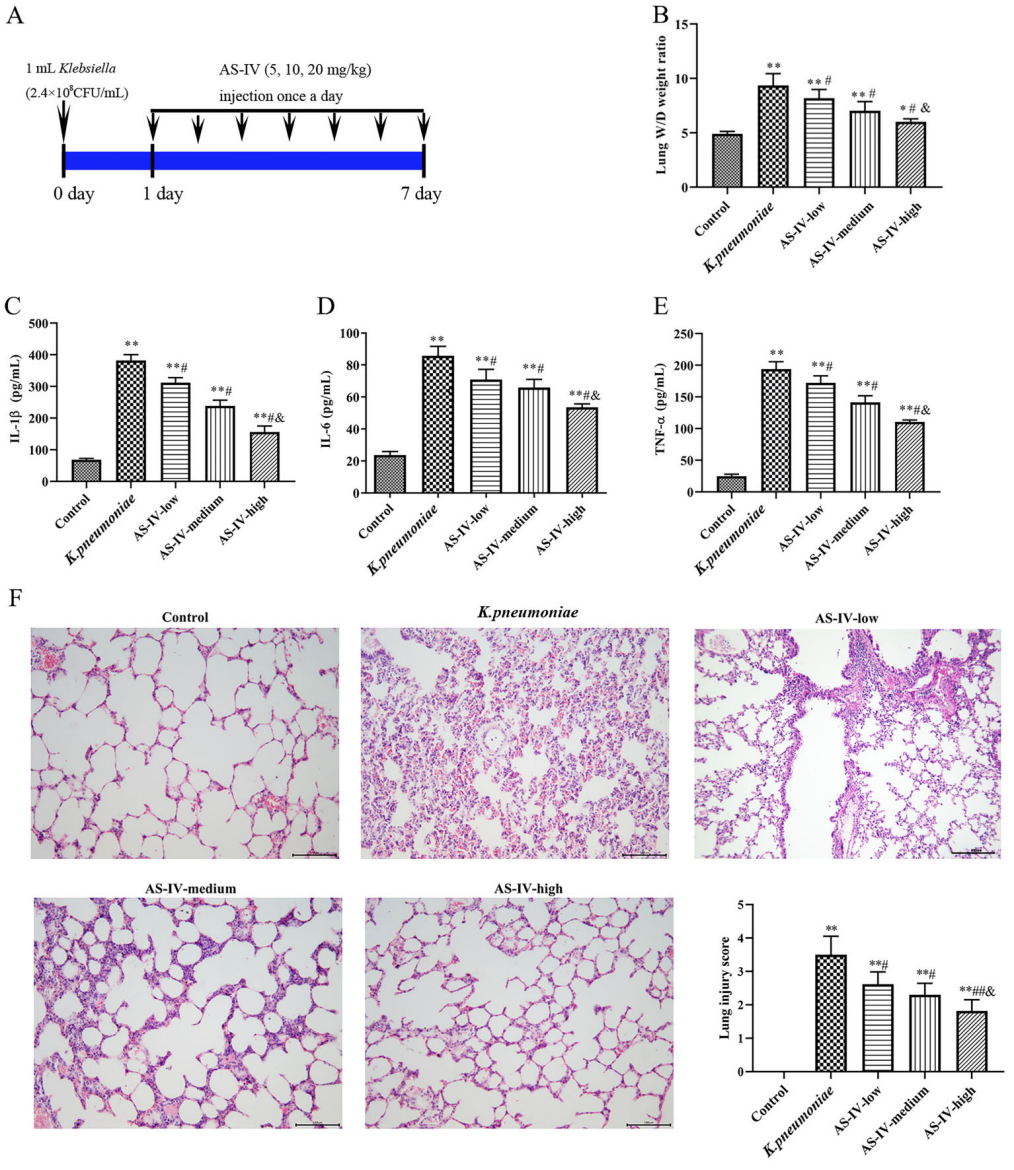
**A**, Diagram of experimental protocols in *K. pneumoniae* rat model treated with astragaloside IV (AS-IV). **B**, The wet/dry (W/D) weight ratios of lung tissues of each group. **C**, The expression of interleukin (IL)-1β, of IL-6 (**D**), and of tumor necrosis factor (TNF)-α (**E**) in bronchoalveolar lavage fluid (BALF) determined by ELISA. **F**, Pathological changes of lung tissue were analyzed with H&E staining (×200; scale bar 100 μm). Data are reported as means±SD (n=5). *P<0.05, **P<0.01 compared with Control group; ^#^P<0.05, ^##^P<0.01 compared with *K. pneumoniae* group; ^&^P<0.05 compared with AS-IV-low group (ANOVA).

The *K. pneumoniae* model was established according to previous research (22). Rats were anesthetized with 3% sodium pentobarbital by intraperitoneal injection before intubation. On the fourth day, 1 mL of *K. pneumoniae* (K46114, National Institutes for Food and Drug Control, China) at a concentration of 2.4×10^8^ CFU/mL was injected into the lung of the rat by endotracheal intubation, while rats of the control group were given an equal amount of 0.9% saline. The model is described in Figure 1A.

### Drug treatment

#### Design 1

To investigate the effects of astragaloside IV (CAS: 84687-43-4, CAT No. S31401, Yuanye, China), rats were randomly divided into 5 groups of 15 rats each: 1) normal control group (Control); 2) *K. pneumoniae* group (*K. pneumoniae*); 3) low astragaloside IV dosage-treated group (AS-IV-low); 4) medium astragaloside IV dosage-treated group (AS-IV-medium); 5) high astragaloside IV dosage-treated group (AS-IV-high).

Astragaloside IV was dissolved in 0.1% dimethyl sulfoxide (DMSO). The drug was given by intragastric administration from the second day of modeling, and the dosages of the drug were in accordance with a previous study (23). According to previous reports (24,25), the 0.1% DMSO dose does not affect lung parameters. Rats in the control group were given 0.1% DMSO by intragastric administration once a day. Rats in the AS-IV-low group were given 5 mg/kg astragaloside IV by intragastric administration once a day, while rats in AS-IV-medium group were given 10 mg/kg, and rats in AS-IV-high group were given 20 mg/kg once a day. Rats were given astragaloside IV for 7 days before further experiments were conducted.

#### Design 2

To further confirm that astragaloside IV was capable of improving *K. pneumoniae* lung injury via regulating TGF-β1/Smad pathway, TGF-β1 inhibitor SB-431542 (CAS: 301836-41-9, MedChemExpress, China) was used to treat rats. Specifically, rats were given 4.2 mg/kg SB-431542 by intraperitoneal administration once a day from the second day of modeling and continued for 7 days (26).

### Enzyme linked immunosorbent assay (ELISA)

Rats were anesthetized by intraperitoneal injection of 3% pentobarbital before endotracheal intubation. Bronchoalveolar lavage fluid (BALF) was obtained by infusing 1 mL of precooled phosphate buffer saline (PBS) and draining the liquid, three times for each rat. The BALF was centrifuged at 4°C (400 *g*, 15 min), and the supernatant was collected. The expression levels of interleukin (IL)-1β (Z02978-1, Genescript, China), tumor necrosis factor (TNF)-α (C008, Novoprotein, China), and IL-6 (CG008, Novoprotein) in BALF were determined with ELISA kits.

### Dry/wet weight ratio of lung tissue

According to previous reports (22), the right lung of the rat was removed from the chest and the wet weight was determined. The dry weight was obtained after the lung has been dried at 80°C for 48 h. Pulmonary edema was assessed by calculating the wet/dry (W/D) weight ratio.

### Hematoxylin-eosin (H&E) staining

Lung tissue was fixed in 4% paraformaldehyde (M13405, Meryer, China) for 24 h before embedding in paraffin and cutting into 5-µm thick slices. These paraffin slices were dewaxed with dimethylbenzene and hydrated with a series of alcohol solution (5 min absolute alcohol, 2 min 95% alcohol, 2 min 80% alcohol, 2 min 70% alcohol). Hydrated slices were stained with H&E (G1120, Solarbio, China) for 15 min and then differentiated with 0.5% hydrochloric alcohol for 30 s. The slices were then soaked in hematoxylin at 50°C for 5 min and stained with eosin for 40 s. Stained slices were washed, dehydrated by alcohol solutions, and clarified with dimethylbenzene. Finally, the slices were observed under an optic microscope (DM1000LED, Leica, Germany).

### Immunohistochemistry

Lung tissue was fixed in 4% paraformaldehyde for 24 h before embedding in paraffin and cut into 5-µm thick slices for immunohistochemistry. Slices were dewaxed with dimethylbenzene and hydrated in a series of alcohol solutions as stated above. Then, the slices were inactivated with 3% H_2_O_2_ methanol solution for 12 min and treated with citrate buffer (pH6.0, M053201, Mreda, China) at 95°C for 10 min. Treated slices were blocked with 5% bovine serum albumin (BSA) for 20 min after cooling down to room temperature. Primary antibody rabbit anti-TGF-β1 polyclonal antibodies (1:100, SAB4502954, Sigma-Aldrich, China) were dropped onto and incubated with slices overnight at 4°C. Slices were washed with PBS for 5 min 3 times and then incubated with PBS-diluted horseradish peroxidase-labelled goat anti-rabbit IgG (1:800, K0034G-AF594, Solarbio) at room temperature. Then, the slices were developed with DAB (SW1020, Solarbio) for 10 min, and counterstained with hematoxylin for 2 min. Stained slices were then differentiated in hydrochloride alcohol solution for 2 s, hydrated with gradient alcohol solutions, clarified with dimethylbenzene, and mounted with neutral resin. Slices were observed under an optic microscope (×200). The gray intensity of the same region of slices under the same condition was analyzed with ImageJ software (version 6; National Institutes of Health, USA).

### Western blot

Lung tissue was lysed with cold RIPA buffer (R0010, Solarbio) containing protease and phosphatase inhibitors on ice for 15 min. The lysate was centrifuged at 12000 *g* at 4°C for 25 min, and the total protein was extracted with a protein extraction kit (BC3640-50T; Solarbio). Then, 40 µg protein sample was separated with 10% SDS-PAGE (Bio-Rad Laboratories, Inc., USA), transferred onto a PVDF membrane (EMD Millipore, USA), and blocked with 5% skim milk at 4°C for 1 h. The membranes were incubated with primary antibodies diluted with 5% BSA at 4°C overnight. The primary antibodies were: TGF-β1 polyclonal antibodies (1:800, SAB4502954, Sigma-Aldrich), p-Smad2 polyclonal antibodies (1:100, orb451161, Biorbyt, China), Smad2 polyclonal antibodies (1:100, orb507656, Biorbyt), p-Smad3 polyclonal antibodies (1:100, orb313112, Biorbyt), Smad3 polyclonal antibodies (1:100, orb94696, Biorbyt), NF-κB p-p65 polyclonal antibodies (1:100, orb501839, Biorbyt), NF-κB p65 polyclonal antibodies (1:100, orb344389, Biorbyt), IκBα polyclonal antibodies (1:1000, 4812, Cell Signaling Technology, China), p-IκBα polyclonal antibodies (1:800, 2859, Cell Signaling Technology), and GAPDH polyclonal antibodies (1:1000,5174, Cell Signaling Technology). Then, the membrane was washed 3 times with TBS-0.01% Tween-20 (TBST), 10 min each time. The membrane was incubated with secondary antibody horseradish peroxidase-labelled goat anti-rabbit IgG (1:1000, K0034G-AF594, Solarbio) at room temperature for 1 h and then washed 3 times with TBST. Bands of proteins were visualized with ECL chemiluminescence reagent (GE2301, Genview, China). The expression levels of proteins were quantified with the ImageJ software.

### Statistics

Data were analyzed with SPSS 20.0 statistical analysis software (IBM, USA), and the data are reported as mean±SD. Data from multiple groups were compared with analysis of variance (ANOVA), while further analysis was accomplished by Tukey test. Statistical significance was indicated by P<0.05.

## Results

### Protective effects of astragaloside IV on *K. pneumoniae* rat

As shown in Figure 1B, there was a significant increase of lung W/D weight ratio in the *K. pneumoniae* group compared with the control group (P<0.01). Compared with the *K. pneumoniae* group, lung W/D weight ratio was decreased by astragaloside IV treatment. The lung W/D weight ratio decreased as the dose of astragaloside IV increased. Compared with AS-IV-low group, there was a significant decrease of W/D weight ratio in AS-IV-high group (P<0.05). The levels of IL-6, IL-1β, and TNF-α in the *K. pneumoniae* group were all significantly higher than the control (P<0.01) (Figure 1C-E). When the pathological changes of lung tissue were further inspected (Figure 1F), there were many inflammatory cells infiltrated in the lung tissue in the *K. pneumoniae* group, resulting in a higher lung injury score, which was significantly reduced by astragaloside IV in a dose-dependent manner (P<0.05). This evidence demonstrated that astragaloside IV had protective effects on *K. pneumoniae*-induced acute lung injury.

### Influence of astragaloside IV on the level of TGF-β1 in *K. pneumoniae* injured rat lung

**Figure 2:**
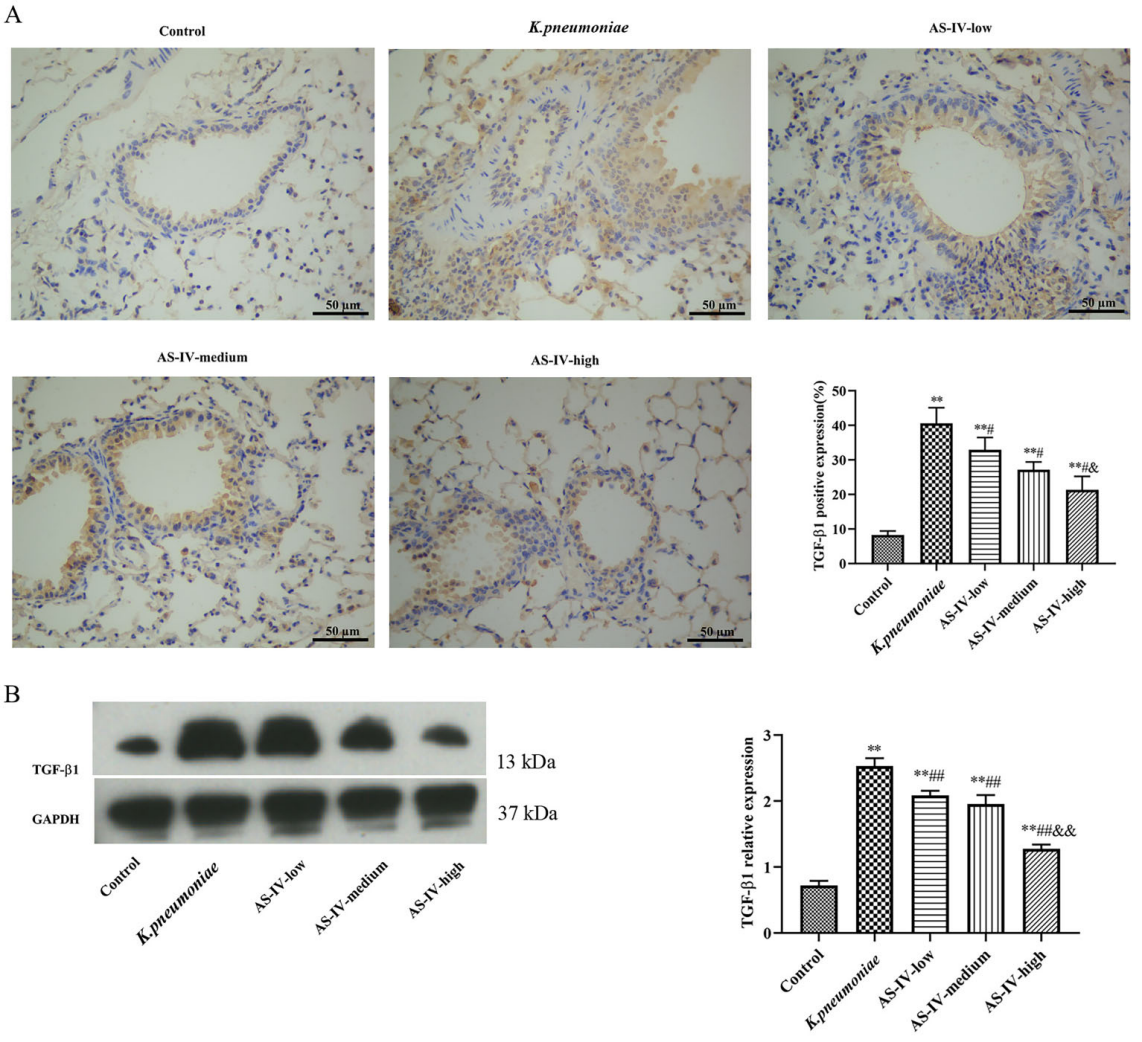
**A**, Expression pattern of transforming growth factor (TGF)-β1 in lung tissues of *K. pneumoniae* rat model treated with astragaloside IV (AS-IV) analyzed by immunohistochemistry (×200; scale bar 50 μm). **B**, Expression pattern of TGF-β1 in lung tissues of each group analyzed by western blot. Data are reported as means±SD (n=5). *P<0.05, **P<0.01 compared with Control group; ^#^P<0.05, ^##^P<0.01 compared with *K. pneumoniae* group; ^&^P<0.05, ^&&^P<0.01 compared with AS-IV-low group (ANOVA).

Rats in the *K. pneumoniae* group expressed much higher TGF-β1 than the control (P<0.05). The expression of TGF-β1 was significantly reduced by astragaloside IV compared with the *K. pneumoniae* group (P<0.05). In addition, the expression level of TGF-β1 in the AS-IV-high group was significantly lower than that in the AS-IV-low group (P<0.05). These results suggested that astragaloside IV might protect the function of the injured lung by regulating TGF-β1 (Figure 2A and B).

### Influence of astragaloside IV on the Smad and NF-κB pathways in *K. pneumoniae* rat

**Figure 3:**
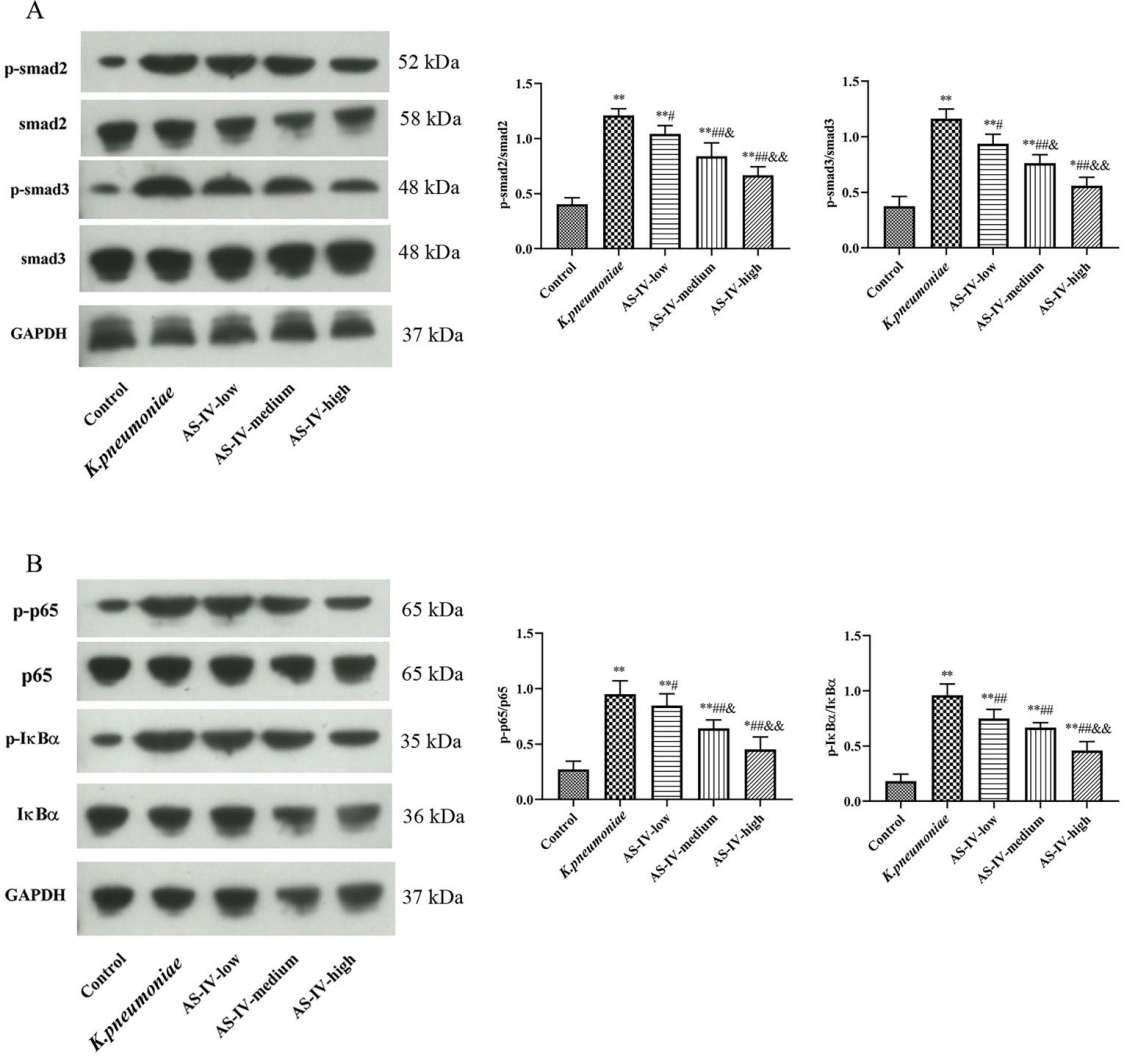
Expression levels of (**A**) Smad pathway- and (**B**) NF-κB pathway-related proteins in *K. pneumoniae* rat model treated with astragaloside IV (AS-IV) determined by western blot. Data are reported as means±SD (n=3). *P<0.05, **P<0.01 compared with Control group; ^#^P<0.05, ^##^P<0.01 compared with *K. pneumoniae* group; ^&^P<0.05, ^&&^P<0.01 compared with AS-IV-low group (ANOVA).

The expression levels of proteins related with the Smad pathway (Figure 3A) and the NF-κB pathway (Figure 3B) in lung tissue were determined by western blot. The levels of p-Smad2/Smad2, p-Smad3/Smad3, p-p65/p65, and p-IκBα/IκBα in the *K. pneumoniae* group were all significantly higher than in the control group (P<0.01), which were evidently reduced by astragaloside IV treatment (P<0.05). Moreover, the effect on the Smad and NF-κB pathways in the AS-IV-high group were significantly greater than in the AS-IV-low group (P<0.05). These results suggested that astragaloside IV might protect rats from *K. pneumoniae*-induced acute lung injury by regulating TGF-β1/Smad pathway thus reducing inflammation.

### Astragaloside IV, as well as a TGF-β1 inhibitor, protected rats from *K. pneumoniae*

**Figure 4:**
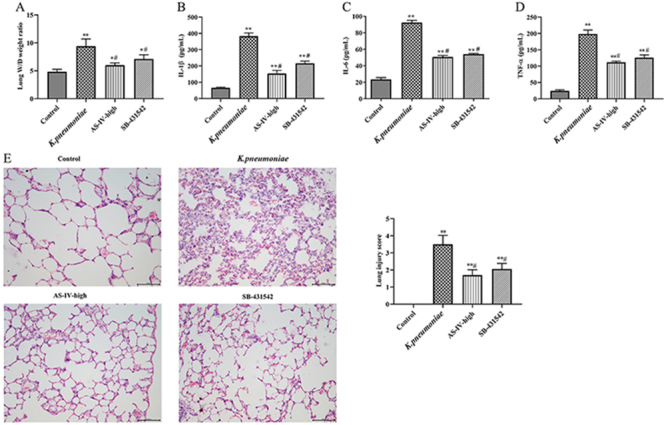
**A**, Lung tissue wet/dry (W/D) weight ratios of *K. pneumoniae* rat model treated with astragaloside IV (AS-IV) or TGF-β1 inhibitor SB-431542. **B**-**D**, Expressions of interleukin (IL)-1β, IL-6, and tumor necrosis factor (TNF)-α in bronchoalveolar lavage fluid (BALF) determined by ELISA. **E**, Pathological changes of lung tissue analyzed with H&E staining (×200; scale bar 100 μm). Data are reported as means±SD (n=5). *P<0.05, **P<0.01 compared with Control group; ^#^P<0.05 compared with *K. pneumoniae* group (ANOVA).

As shown in Figure 4A, the W/D lung tissue weight ratios of the AS-IV-high group and the SB-431542 group were significantly reduced compared with the *K. pneumoniae* group (P<0.05). The levels of IL-6, IL-1β, and TNF-α in BALF in the *K. pneumoniae* group were all significantly higher than in the other groups (P<0.05) (Figure 4B-D). However, levels of those pro-inflammatory cytokines were significantly decreased after the high dose of astragaloside IV or SB-431542 treatment (P<0.05). In addition, there was severe infiltration of inflammatory cells in the lung tissue of rats in the *K. pneumoniae* group, which was reversed by the high dosage of astragaloside IV or SB-431542 treatment, thus reducing the lung injury score (P<0.05) (Figure 4E). These data demonstrated that astragaloside IV and a TGF-β1 inhibitor were capable of protecting rats from *K. pneumoniae*-induced acute lung injury.

### Astragaloside IV and a TGF-β1 inhibitor regulated Smad pathway in lung injury

**Figure 5:**
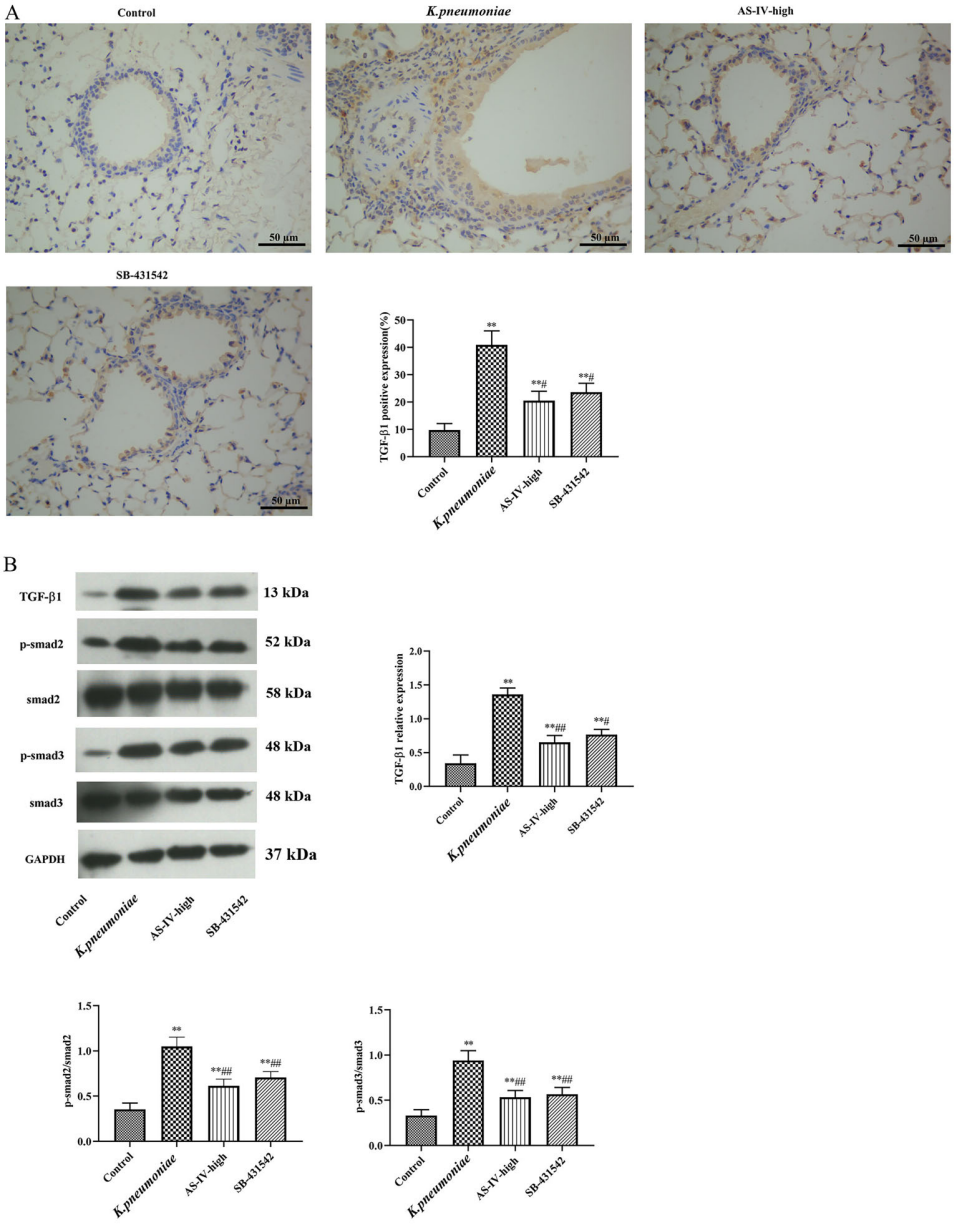
**A**, Expression of transforming growth factor (TGF)-β1 in lung tissues in *K. pneumoniae* rat model treated with astragaloside IV (AS-IV) or TGF-β1 inhibitor SB-431542 detected by immunohistochemistry (scale bar 50 μm); **B**, Expression levels of proteins related with TGF-β1/Smad, p-Smad2/Smad2, and p-Smad3/Smad3 pathways in lung determined by western blot. Data are reported as means±SD (n=3). *P<0.05, **P<0.01 compared with Control group; ^#^P<0.05, ^##^P<0.01 compared with *K. pneumoniae* group (ANOVA).

The level of TGF-β1 decreased after 20 mg/kg astragaloside IV or SB-431542 treatment compared with the *K. pneumoniae* group (P<0.05) (Figure 5A and B). In addition, treatment of 20 mg/kg astragaloside IV or SB-431542 also reduced the levels of p-Smad2/Smad2 and p-Smad3/Smad3, which were increased by *K. pneumoniae* (P<0.05) (Figure 5B). These data suggested that astragaloside IV and a TGF-β1 inhibitor could regulate Smad pathway in lung injury.

### Astragaloside IV and a TGF-β1 inhibitor regulated NF-κB pathway in lung injury

**Figure 6:**
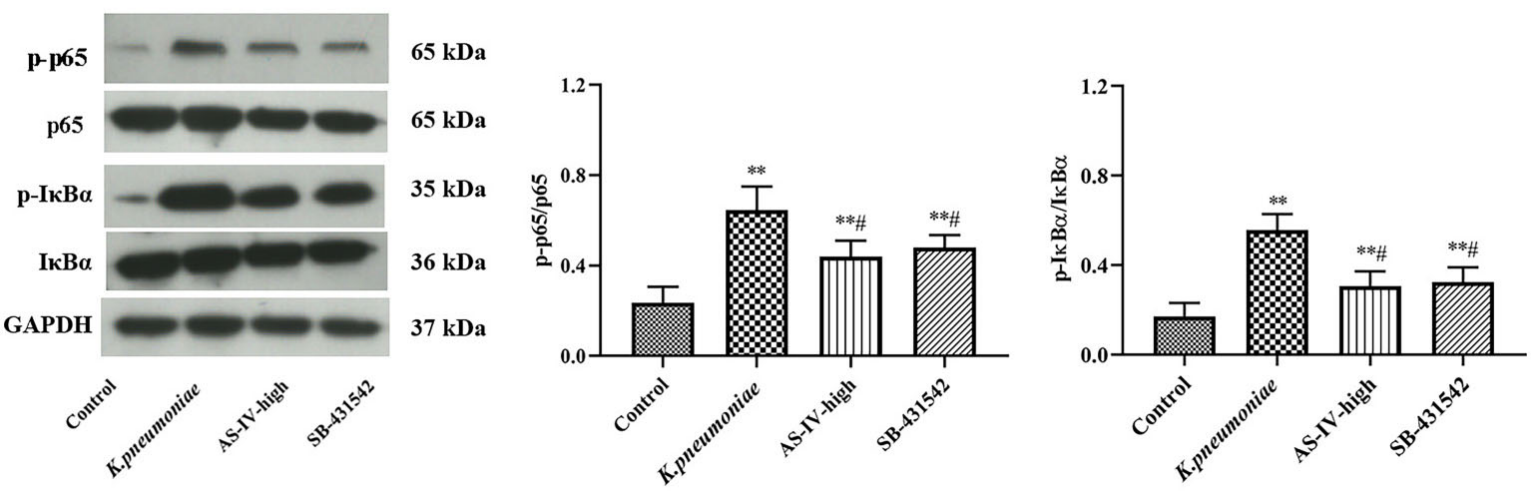
Expression levels of proteins related to NF-κB pathway in lung in *K. pneumoniae* rat model treated with astragaloside IV (AS-IV) or TGF-β1 inhibitor SB-431542 determined by western blot. Data are reported as means±SD (n=3). *P<0.05, **P<0.01 compared with Control group; ^#^P<0.05 compared with *K. pneumoniae* group (ANOVA).

The expression levels of p-p65/p65 and p-IκBα/IκBα in lung tissue were significantly reduced by 20 mg/kg astragaloside IV treatment compared with the *K. pneumoniae* group (P<0.05) (Figure 6). The results suggested that astragaloside IV could also regulate NF-κB pathway in lung injury, as did the TGF-β1 inhibitor.

## Discussion

*K. pneumoniae* rat model, based on which further experiments were performed, was successfully established according to a former research article (22). *K. pneumoniae* induces great damage in the lung of rats, including inflammation, edema, and cell death, which could be alleviated by astragaloside IV treatment in a dose-dependent manner.

It has been reported that astragaloside IV alleviates pulmonary fibrosis via inactivation of TGF-β1/Smad signaling pathway, so the expression level of TGF-β1 in lung was inspected by immunohistochemistry and western blot (15,17). The results showed that astragaloside IV reduced TGF-β1 expression induced by *K. pneumonia* infection, which was consistent with former research that reported the TGF-β1 inhibiting effect of astragaloside IV (27,28).

To further study the relationship between astragaloside IV and TGF-β1/Smad pathway in *K. pneumoniae* rats, the levels of proteins related with the Smad pathway and NF-κB pathway were determined by western blot. The results showed that astragaloside IV could reduce the phosphorylation levels of Smad2, Smad3, p65, and IκBα that were induced by *K. pneumonia* infection, indicating the involvement of the Smad pathway and NF-κB pathway. It has been well-known that TGF-β receptors are able to phosphorylate members of the Smad family, especially Smad2/3, to exert biological functions after activation by TGF-β (29). Several pro-inflammatory cytokines including IL-6, IL-1β, and TNF-α were also decreased after astragaloside IV treatment, indicating that astragaloside IV could inhibit inflammation-related apoptosis caused by *Klebsiella* infection by inhibiting TGF-β1 signaling pathway. In addition, the involvement of NF-κB pathway was also observed in this study. The phosphorylation levels of NF-κB key component p65 and the inhibitory factor IκBα were all decreased after astragaloside IV treatment, which would contribute to the inhibition of the NF-κB pathway (30,31). The effect on NF-κB signaling pathway might be explained by the coordination between TGF-β1/Smad and NF-κB pathway, as reported formerly (32). Thus, this study demonstrated that astragaloside IV, as well as a TGF-β1 inhibitor, was able to alleviate lung injury caused by *K. pneumoniae*, which affected downstream effectors including Smad2/3 and pro-inflammatory cytokines as reported previously, and the NF-κB pathway was also involved in the process (33).

To further confirm the effect of astragaloside IV on TGF-β1 in this disease model, TGF-β1 inhibitor SB-431542 was used in the following experiments, which was comparable to the effects of the high dosage of astragaloside IV at both tissue and molecular levels, though the protective effects of high dosage astragaloside IV were slightly higher. The results suggested that both astragaloside IV and SB-431542 were capable of recovering lung tissue damage caused by *K. pneumoniae* by inhibiting TGF-β1/Smad and NF-κB pathways. Based on the multi-target nature of traditional Chinese medicine and the subtle difference between the two compounds, the existence of a potential unknown molecular pathway controlled by astragaloside IV other than the TGF-β1/Smad signaling pathway (34,35) could be easily hypothesized.

It would be very important to identify the direct targets of astragaloside IV to understand the molecular mechanisms of the drug in detail for better treatment and fewer side effects. Traditional Chinese medicine, especially herbs, are regarded as a treasure reservoir to deal with infections caused by various pathogens, including COVID-19 (36). Astragaloside IV, a compound that is mainly extracted from the roots of herbal plants, was found to be capable of alleviating *K. pneumoniae*-induced lung injury for the first time in this study.

However, the effects of astragaloside IV treatment on lung colony-forming units (CFU) were not studied in this research, which is the limitation of this work. Whether astragaloside IV could reduce the bacterial load is crucial to *K. pneumoniae* infection. In further studies, pulmonary CFU will be assessed in *K. pneumoniae* infection.

In summary, astragaloside IV alleviated acute lung injury of rats caused by *K. pneumoniae* through inhibition of TGF-β1/Smad signaling pathway. Although the molecular mechanism of TGF-β1 inhibition by astragaloside IV remains unknown, findings of this study revealed the protective effects of astragaloside IV in a *K. pneumoniae* model, which is of great importance to provide clues for treatment of this hospital-acquired infection.
